# The Antioxidants Changes in Ornamental Flowers during Development and Senescence

**DOI:** 10.3390/antiox2030132

**Published:** 2013-08-06

**Authors:** Marina Cavaiuolo, Giacomo Cocetta, Antonio Ferrante

**Affiliations:** Department of Agricultural and Environmental Sciences, Università degli Studi di Milano, via Celoria 2, Milano 20133, Italy; E-Mails: marina.cavaiuolo@unimi.it (M.C.); antonio.ferrante@unimi.it (A.F.)

**Keywords:** antioxidant, edible, flower, ornamentals, senescence

## Abstract

The concentration of antioxidant compounds is constitutive and variable from species to species and is also variable considering the development of the plant tissue. In this review, we take into consideration the antioxidant changes and the physiological, biochemical and molecular factors that are able to modulate the accumulation of antioxidant compounds in ornamental flowers during the whole development process until the senescence. Many ornamental flowers are natural sources of very important bioactive compounds with benefit to the human health and their possible role as dietary components has been reported. The most part of antioxidants are flower pigments such as carotenoids and polyphenols, often present in higher concentration compared with the most common fruits and vegetables. The antioxidants content changes during development and during senescence many biochemical systems and molecular mechanisms are activated to counteract the increase of reactive oxygen species and free radicals. There is a tight correlation between antioxidants and senescence processes and this aspect is detailed and appropriately discussed.

## 1. Introduction

The source of nutraceutical compounds in human diet is almost exclusively provided by fruits and vegetables. However, flowers are becoming important sources of several bioactive compounds that can be added in the diet as food. In the ancient time flowers were mainly eaten for their medicinal properties rather than their nutritional value. Nowadays, several metabolomics studies revealed the chemical compositions of wild and ornamental flowers, showing the presence of important bioactive molecules. Often, wild flowers represent low cost sources of important natural antioxidants. However, edible flowers are also used by chefs to add color, fragrance and flavor to foods or drinks [1]. In the past, the relative low interest in the use of ornamental flowers as food sources was due to the limited analytical methods able to detect poison substances; ornamental flowers have been largely used as sources of natural poison and this explains why many common flowers used as garden decoration are poisonous.

The majority of edible flowers are vegetables such as pumpkins flowers or inflorescences (broccoli) and a lot of references on their chemical composition or nutritional values are available. Several species of ornamental flowers are also substantial sources of antioxidants able to remove the negative effect of free radicals. In humans, antioxidants exert important roles in preventing several degenerative diseases and stress related pathologies. The intake of antioxidant molecules can scavenge free radical species to defend the cells from damages. In flowers, important compounds with antioxidant activities and anti-inflammatory proprieties are represented by polyphenols, carotenoids and vitamin C (ascorbic acid) [2]. Polyphenols include more than 10,000 compounds and are among the most important natural antioxidant compounds [3]. Flowers with their pigments are rich in phenolics which include phenolic acids, flavonoids and anthocyanins. From an ecological point of view the flowers represent the most important organs for the plants, since they are responsible for the spatial and temporal species survival. The flower has to be protected during development in order to guarantee the fertilization. After pollination, the external parts of flowers such as petals or tepals, stamens and part of the pistil, such as style and stigma, senesce because they have completed their function.

Therefore, the variations in the levels of antioxidant compounds are related with floral developmental processes and especially with the last step of senescence. The plant defense mechanisms against external and internal senescence associated factors are extremely complex. Multidisciplinary studies using physiological, biochemical and molecular biology approaches should be able to give a clearer picture of this fascinating phenomena and its regulation.

This review focuses on the use of ornamental flowers as food and source of antioxidant compounds as well as on the molecular mechanisms of flower development and floral senescence in general. In particular, the role of antioxidant compounds, the function of senescence associated genes (SAGs), antioxidant genes and their regulatory networks from signal transduction to transcription and post-transcription are discussed. Flower development has been largely studied for understanding the biological processes linked with senescence. Since flowers constitute floriculture items, it is very important to study the function of factors involved in flower senescence to identify innovative strategies for preserving the ornamental quality during storage, prolong the flower life or vase life at the consumer stage and increase the antioxidant compound levels.

## 2. Antioxidants in Ornamental Flowers

Flowers are appreciated for their wide range of colors and aroma. These sensory attributes are valuable indicators of the external quality and the ornamental value as well as important properties used for culinary purpose. Many ornamental flowers contain high levels of antioxidant compounds, which are often even higher than common horticultural crops. One of the most important parameters used to estimate the antioxidant content is the determination of the total phenolics. Flowers with higher total phenolics content are *Antigonon leptopus*, *Bougainvillea glabra*, *Tagetes erecta*, *Cosmos sulphureus*, *Prunus mume* and *Sophora viciifolia* with values >100 mg/g DW (Table 1). Several analytical methods can be used to evaluate the antioxidant capacity of flower tissues such as 2,2-diphenyl-1-picrylhydrazyl (DPPH) and Ferric-reducing antioxidant power (FRAP). The higher values of FRAP were found in *Antigonon leptopus*, *Bougainvillea hybrid*, *Cosmos sulphureus*, *Nelumbo nucifera* and *Tagetes erecta* (Table 1). With respect to fruit or vegetables such as apple and lettuce, all the species studied as edible flowers have higher antioxidant capacity expressed as FRAP and lower percentage of inhibition expressed as DPPH.

**Table 1:** Total phenolic content (determined with Folin–Ciocalteu assay) and antioxidant capacity (FRAP and ORAC assay) in different edible flowers.

Edible flower	Total phenolics		Antioxidant capacity (DPPH)		Antioxidant capacity (FRAP)	Ref.
	mg/g DW	mg/g FW	g AsA equiv./kg FW	% inhibition	μmol Fe^2+^/g DW	
*Antigonon leptopus*	72.1–177.2			89.4	282.9–619.7	[4]
*Antirrhinum majus*		3.49	5.06		-	[5]
*Begonia boliviensis*		4.92	6.80		-	[5]
*Bougainvillea glabra*	138.2				307.1	[4]
*Bougainvillea hybrid*	50.0			91.4	588.0	[4]
*Centaurea cyanus*		4.76	6.81		-	[5]
*Chrysanthemum frutescens*		2.53	4.24		-	[5]
*Chrysanthemum parthenium*		2.72	4.21		-	[5]
*Clitoria ternatea*	59.0		-		73.0	[4]
*Cassia siamea*	88.5			97.6	163.7	[6]
*Cosmos sulphureus*	86.8–102.5			87.0	99.9–538.6	[4]
*Dianthus caryophyllus*		5.28	6.96			[5]
*Fuchsia x hybrid*		3.45	5.20			[5]
*Helianthus annuus* L.	47.1					[7]
*Hemerocallis* spp.		69–160				[8]
*Impatiens walleriana*		4.85	6.89			[5]
*Ixora chinensis*	82.4					[4]
*Malvaviscus arboreus*	59.0			31.4	271.3	[4]
*Nelumbo nucifera*	60.0		96.9		585.4	[4]
*Plumeria obtusa*	37.0		69.6		260.3	[4]
*Prunus mume*	150					[4]
*Rosa odorata*		5.02	6.85			[5]
*Rosa hydrida*		8.5				[9]
*Sophora viciifolia*	143.8			20.7	3160	[10]
*Tagetes erecta*	98.0–212.9		94.3		329.4–609.2	[4]
*Tagetes patula*		4.58	6.70			[5]
*Telosma minor*	29.0			34.1	162.6	[4]
*Tropaeolum majus*		3.31	5.12			[5]
*Viola x wittrockiana*		5.11	6.65			[5]
*Malus domestica*	100–200	1.2–6.5	83		4.2–6.3	[11]
*Lactuca sativa*		0.2–0.3		74–82	1.8–5.3	[12]

Among the flavonoids, quercetin was present in high concentrations. In *Nelumbo nucifera* and *Plumeria obtuse* this flavonoid reached values of 194–238 mg/100 g DW. In contrast, rutin was present at concentration over 50 mg/100 g DW in *Bougainvillea hybrid*, *Ixora chinensis* and *Plumeria obtuse*. In apple and lettuce this compound is usually not detectable. Quercetin and rutin are able to protect human health against diseases associated to oxidative stresses such as cancer and cardiovascular diseases [13]. The daily dose intake of quercitin should range from 10 to 20 mg. Another important flavonoid with beneficial effects on human health is kaempferol. *In vitro* assay showed the ability of kaempferol to affect positively several cancer-derived disorders such as apoptosis, angiogenesis, metastasis, and inflammation [14].

The flowers with higher content of kaempferol were *Antigonon leptopus*, *Bougainvillea glabra* and *Tagetes erecta*, ranging from 76 to 87 mg/100 g DW (Table 2).

**Table 2:** Flavonoid compounds in different edible flowers expresses as mg/100 g DW, apple and lettuce were added for comparison. Data are means found in literature.

Species	Apigenin	Catechin	Chlorogenic acid	Kaempferol	Myricetin	Quercetin	Rutin	Ref.
*Antigonon leptopus*	0.83			75.9	47.5		5.7–21.9	[15]
*Bougainvillea glabra*	8.9			87.2	61.5		1.3	[15]
*Bougainvillea hybrida*	-			3.54	5.6		51.5	[4]
*Cassia siamea*	-	-	-	3.21	4.56	61.9	64	[4]
*Cosmos sulphureus*	7			25.6	60		19.7	[15]
*Hemerocallis* spp.	-	111.5	7.2			9	14.6	[16]
*Ixora chinensis*	0.64	-	-	3.77	5.18	102.4	139	[4]
*Leucaena leucocephalade*	-	-	-	4.23	5.72	67.1	16.2	[4]
*Malvaviscus arboreus*	-	-	-	3.18	5.05	33.6	27.7	[4]
*Nelumbo nucifera*	0.62	-	-	3.79	5	237.8	23.1	[4]
*Plumeria obtuse*	-	-	-	3.58	5.06	193.6	500.3	[4]
*Tagetes erecta*	8.4			83.4	54.8		5.1	[15]
*Malus x domestica*	-	38.8–99.3	75.1	3.1	30.9	7.7–13.20	82	[17,18,19,20,21]
*Lactuca sativa*	<4	nd	47	2.9	<1	42.9	nd	[22,23]

In apple kaempferol is under the detection limits, while in lettuce it is comparable with other flower.

The antioxidant compounds increase during early senescence and decline during advanced senescence. As a first reaction, antioxidant compound protect the cell from damaging substances such as hydrogen peroxide and other reactive oxygen species (ROS).

### 2.1. The Changes of Antioxidant Compounds Is Often Related to the Senescence Processes

The antioxidant compounds increase during early senescence and decline during advanced senescence. As a first reaction, antioxidant compound protect the cell from damaging substances such as hydrogen peroxide and other reactive oxygen species (ROS).

Senescence is a process of progressive oxidative deterioration that represents the last step in flower development and leads ultimately to the programmed cell death (PCD). ROS are a common feature in the life of aerobic organisms. They act as signal molecules and are involved in several physiological processes in both plants and animals. Senescence is a complex chain of phenomena and ROSs are responsible of several senescence related processes. In fact, a certain amount of ROS is always produced during the whole life of each aerobic organism and its levels are tightly controlled by antioxidant systems which involve the mutual action of enzymes and antioxidant compounds.

The onset of senescence requires the coordinated regulation of several genes which is triggered by internal and external factors. The plant hormones ethylene and abscisic acid play an important role as internal signals of flower senescence. Based on the response to ethylene, flowers are classified in ethylene sensitive or ethylene insensitive. In the first class, ethylene strongly regulates the senescence process. In some species such as Hibiscus (*Hibiscus rosa-sinensis* L.) both hormones are involved in the flower senescence [24].

Plants counteract the effects of oxidative stress by synthesizing new antioxidants, by utilizing pre-existing pools or activating specific routes to recycle and consume specific molecules. A challenge for future researches is to understand if senescence could stimulate the *de novo* synthesis of antioxidant compounds or if the antioxidant potential of flowers during senescence mainly depends on the pool of molecules already accumulated during flower development.

#### 2.1.1. Ascorbic Acid (AsA)

The AsA represents a key molecule in plant metabolism. It has been recognized to play a central role in several physiological processes like photosynthesis, photo-protection, cell division, plant growth, stress responses, regeneration of other important molecule such as α-tocopherol. Moreover, it has been reported to act as a co-substrate for the biosynthesis of important plant hormones such as ethylene and gibberellic acid and to be the substrate for oxalate and tartrate biosynthesis [25,26,27].

The AsA has been reported to be involved in the process of cell wall metabolism [28] and cell wall oxidative scission [29]. Further studies are needed in order to clarify the complex net of correlations between the levels of AsA, cell wall polysaccharides, ROS and other antioxidant molecules.

The AsA was reported to be involved in regulation of some senescence associated genes (SAGs) during plant oxidative stress-induced senescence. In fact a study showed an up-regulation of specific SAG transcripts in the AsA-deficient *Arabidopsis* mutant *vtc1* grown under a long-day photoperiod (16 h) [30], suggesting that AsA deficiency induces a senescent phenotype.

It has been suggested that the effects of AsA levels on flowering time could be related to alterations in phytohormone levels such as gibberellic acid (GA) ABA, SA and ethylene and that the redox status of AsA may play a role in signaling in this interconnected phytohormone network. [30]. In daylily (*Hemerocallis hybrid*) petals AsA levels decreased by one half after 12 h before flower opening and the levels remain low to 36 h after flower opening. A limiting amount of AsA has been thus mentioned by authors as an important factor in the ability of ascorbate peroxidase (APX) to reduce H_2_O_2_ [31]. The AsA was reported to rapidly decline during aging of *Chrysanthemum* petals and it was reported to interact synergistically with α-tocopherol in contrasting oxidative damage of lipid membranes [32].

#### 2.1.2. Tocopherol

In plants, tocopherols together with tocotrienols are important antioxidants believed to act as scavenger of lipid peroxy radicals, which are responsible for lipid peroxidation [33,34,35]. The α-tocopherol represents the primary defense against lipid peroxidation, it has been reported to act in preventing radical-induced lipid peroxidation and its action is synergistically connected to AsA [32,36]. In petals from recently cut chrysanthemum flowers the ascorbic acid/α-tocopherol ratio declined during aging, suggesting that this antioxidant system is less efficient as the aging process started [32].

#### 2.1.3. Phenolic Compounds

Phenolic secondary metabolites are widespread among plants and are involved in many plant functions, defense mechanisms and stress responses. Anthocyanins, a flavonoid subclass, are the main pigments in flowers acting as insect and animal attractants [37,38]. Anthocyanins are synthesized via the phenylpropanoid pathway. These compounds are known for their antioxidant properties and are among the most important plant’s antioxidants. They have been extensively studied and their pathway of accumulation has been well characterized [39,40,41].

The flavonols generally increase prior to anthocyanin accumulation during floral development and declined when anthocyanin began to accumulate [42,43]. This phenomenon is well tightly regulated and it was explained, hypothesizing a role for flavonols in protecting developing sexual organs from potentially harmful UV-B light [43,44].

The changes in concentration of various phenolic compounds and color parameters were measured for the first time at different developmental stages of miniature rose “KORcrisett” [9]. In their work, the authors referred to a previous research published by Takahama *et al.* in 1997 [45] in which the existence of a peroxidase/phenolics/ascorbate system active in the scavenging of hydrogen peroxide in plant cells was suggested and reported that a decline in phenolics and anthocyanins concentration at later stages of flower development may limit the role of this system, making the flower more vulnerable to oxidative stress. The authors thus suggested a role of phenolics in regulation of flower development [45]. During flower senescence in long life and ephemeral *Hibiscus* (*Hibiscus rosa-sinensis* L.) anthocyanins were very high in ephemeral flowers and were reported to be likely linked to senescence processes [24,46]. In petunias the flower longevity was inversely correlated to anthocyanins content [47]. On the contrary, other studies showed a negative correlation between high antocyanins content and flower longevity. In senescent *Orchid* flowers changes in the anthocyanin pattern determined color changes from white to pink or blue [48]. Analogously during flower senescence the anthocyanin accumulation increases and the application of senescence promoters (ethylene) or inhibitor (1-methylciclopropene) enhance or retard the anthocyanin accumulation [49]. These results indicate that phenolic compounds biosynthesis might be a response of flowers to cell disruption, but the accumulation of these phenolic compounds is inefficient to counteract the genetically programmed senescence.

#### 2.1.4. Carotenoids

Carotenoids (in relation to abscissic acid ABA biosynthesis) levels generally decreased during senescence indicating a possible involvement as a substrate in biosynthesis of ABA through the indirect pathway. The highest carotenoids concentration was associated with shortest flower life, suggesting their potential source for ABA biosynthesis in petals. However, carotenoids degradation and ABA content should be monitored during flower development and senescence in all cultivars before to come to a clear conclusion [46].

## 3. Biochemical Pathways Involved in the Antioxidant Responses during Flower Senescence

**Figure 1:**
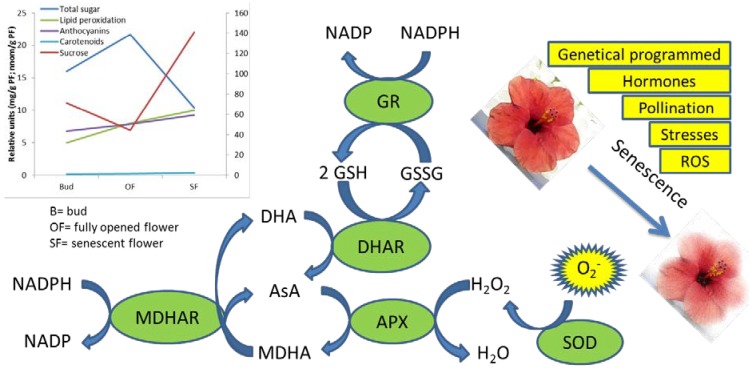
Factors involved in the flower senescence and detoxification enzymes, which acts in scavenging the superoxide forms. In the graph, nutraceutical compounds and senescence markers such as lipid peroxidation during development and senescence are reported.

Senescence is tightly associated with a rise in ROS levels [50], whose production is accompanied by the activation of the enzymes involved in ROS scavenging such as superoxide dismutases (SODs), catalases (CATs) and ascorbate-glutathione cycle enzymes ascorbate peroxidase (APX), monodehydroascorbic reductase (MDHAR), dehydroascorbic reductase (DHAR) and glutathione reductase (GR) (Figure 1). These antioxidant enzymes show different pattern of activity among species in ethylene-dependent and independent manners. The levels of SODs were observed to decrease with the progression of senescence in petals from *Chrysanthemum* [32], *Rose* [51], day lily [52], *Carnation* [53], *Iris* [54], *Gladiolus* [55] and *Freesia* [56]. Class III plant peroxidase (POX), such as peroxiredoxins [57] and glutathione peroxidases [58] were recently identified as being involved in ROS scavenging by reaction with H_2_O_2_ or alkyl hydroperoxides [59]. During senescence CAT activity was shown to fall in *Chrysanthemum* [32], *Gladiolus* [55] and carnation [60], but to rise in day lily [52] and *Iris* [54]. Ascorbate peroxidase (APX) reduces H_2_O_2_ with the consequent oxidation of ascorbate to dehydroascorbate: H_2_O_2_ acts as a signaling molecule to induce PCD pathway and expression of defense-related genes (including glutathione *S*-transferase, glutathione peroxidase, superoxide dismutase) [61]. In *Gladiolus* [62], day lily [52] and *Iris* [54] the down regulation of APX over the senescence period has led to high accumulation levels of endogenous H_2_O_2_ that up-regulates SOD gene action. In contrast, in *Chrysanthemum* and carnation petals, APX activities increase during flower senescence accompanied by an increase in the number of peroxisomes [63,64].

More studies are needed in order to better understand the possible role of MDHAR and DHAR as their activity lead to AsA recycling and thus a better knowledge about the interaction between the activity of these enzyme with AsA levels it could represent an useful mean to reinforce flower’s own ability to counteract the progress of senescence, increasing their marketability and antioxidant quality. The GR activity showed diverse pattern of activity between species during floral senescence. In Gladiolus, GR levels fell at later stages of senescence [62,65]; in carnation, GR declined much earlier throughout senescence [60]; in *Chrysanthemum*, GR peaked twice, at late bud stage and again at the beginning of senescence [63]. All these studies clearly indicated that the activity of many ROS-scavenging enzymes follows a decline as senescence reaches the last phases, in concomitance with a reduction in the antioxidant content.

## 4. The Molecular Regulatory Networks of Flower Development and Senescence

### 4.1. Gene Regulation of Flower Development

Flower formation and development are controlled by many genes, mostly encoding for transcription factors. These genes are conventionally grouped into classes according to their role in the processes of flower initiation, meristem identity and organs determination [66]. In *Arabidopsis*, the class of Flowering Time genes control the flowering time and is regulated by environmental conditions such as cold temperature (vernalization) [67]. The key gene in the control of flowering time is the FLOWERING LOCUS C (FLC), a MADS-box transcription factor whose expression is regulated at the epigenetic level [68]. The repression of FLC target genes lead to a delay in the expression of the Floral Meristem identity genes. This latter class of genes includes: LEAFY (LFY) and APETALA1 (AP1), two transcription factors with partial overlapping roles in the determination of flower meristem identity; CAULIFLOWER (CAL) and UNUSUAL FLORAL ORGANS (UFO) both promoting the floral meristem fate, and TERMINAL FLOWER 1 (TFL1), which maintains the inflorescence meristem identity [69,70,71]. Other genes promoting meristem identity include SEPALLATA (SEP) and other uncharacterized genes that were shown to be target of LFY activity [72]. The class of Flower Organ Identity genes determine the floral-organ patterning of each whorl and include A, B, C, D, and E homeotic genes. The ABCDE model is found to be conserved among several plant species [73,74]. Class A specifies sepal identity in whorl 1 and along with class B determines petal identity in whorl 2; class B along with class C specifies stamen identity in whorl 3 and class C confers carpel identity in whorl 4; class D determines ovule identity and class E is required for petals, stamens and carpels specification [75].

### 4.2. Senescence Associated Genes (SAGs)

Few recent reviews on floral senescence are available [50,76]. Flower senescence is a programmed process characterized, in the terminal phase, by the degradation of nucleic acids, proteins, carbohydrates, lipids and membranes, all representing hallmarks of programmed cell death (PCD) [76]. These events are orchestrated at different levels, including control of metabolic changes, environmental and hormonal signaling in association with considerable modification of gene transcription and translation. The molecular and regulatory networks involved in flower senescence program have been largely investigated: a high number of senescence-associated genes (SAGs) were identified in flowers with different patterns of senescence such as *Petunia*, *Dianthus caryophyllus* and *Hemerocallis* [77,78,79]. Moreover, by means of PCR-based subtractive hybridization, microarray technology and EST analyses numerous genes with up- or down-regulation expression patterns were identified also in *Narcissus* [80], *Alstroemeria* [81], *Iris* [82], *Rosa* [83] and *Mirabilis jalapa* [84]. SAG-encoded proteins exert a variety of functions in macromolecule breakdown, ROS detoxification, ethylene biosynthesis, nutrient recycling and remobilization. SAGs include enzymes involved in lipid metabolism (glutathione-*S*-transferase, allene oxide synthase, acyl CoA oxidase, in-chain fatty acid hydroxylase and fatty acid elongase); hydrolase implicated in cell-wall degradation (β-glucosidase, β-galactosidase, pectin acetylesterase), a carboxy PEP mutase with putative role in membrane turnover and biosynthesis of phosphonates, and enzymes responsible for ethylene biosynthesis (*S*-adenosylmethionine synthetase, 1-aminocyclopropane-1-carboxylic acid synthase (ACC) and 1-aminocyclopropane-1-carboxylic acid oxidase) [85,86,87,88,89,90].

In *Alstroemeria* the down-regulation of a cytochrome P450 was associated with flower senescence [81] while in *Petunia* a tonoplast-localized cytochrome P450 highly similar to a tomato allene oxide synthase (AOS) was reported to be highly expressed in senescing petals [91].

In *Rose*, an homologous gene of a delta-9-desaturases was induced during senescence with putative role in degradation of saturated fatty acids of membrane lipids [92].

Work performed in *Mirabilis jalapa* identified down-regulated genes homologous to clock-associated (CCA1), far-red insensitive 219 (FIN219) and phytochrome A-related WD-40 repeat gene, suggesting that flower senescence might be under the control of the circadian clock through the phytochrome A pathway [93,94]. In addition, a RING zinc finger ankyrin repeat protein (MjXB3) was found highly expressed in senescing petals with putative roles in protein ubiquitination [76]. The involvement of an ubiquitin pathway for the degradation of petal proteins was also reported in *Hemerocallis* flowers [95]. Several cysteine and serine protease genes were shown to be up-regulated in *Narcissus* [96] and under exposure to ethylene in *Petunia* [97], *Sandersonia* [98] and *Alstroemeria* [99]. Thiol proteases (SEN11 and SEN102) were up-regulated in *Hemerocallis* sepals and found to be involved in protein hydrolysis at the late senescence stage [100]. Nine cysteine proteases were identified in senescent corollas of *Dianthus caryophyllus* and their expression was highly increased by ethylene [101] in parallel with the reduction of cysteine protease inhibitor [102].

It was shown that the gene expression levels of nuclease and DNA fragmentation increase until the advanced stages of flower senescence in *Petunia*: in particular, five single-stranded and double-stranded DNases were identified and induced by Ca^2+^ in senescent petals of pollinated flowers [103]. In *Hemerocallis* petals, the S1- and P-type endonucleases were identified with roles in fragmentation of single-stranded DNA and RNA [87]. Senescence-associated S-like RNases were also characterized in the petals and leaves of *Arabidopsis* and tomato [104,105].

All floral tissues undergo senescence. For example, the degeneration of anther tapetum was characterized by chromatin condensation and DNA degradation in *Lobivia rauschii*, *Tillandsia albida* and *Hordeum vulgare* [106,107]. In the cytoplasmic male sterility mutant (PET1-CMS) of *Helianthus annuus* the release of cytochrome c from the mitochondria into the cytoplasma caused premature PCD [108]. In papaver, the PCD deriving from self-incompatibility (SI) was induced by caspase-3-like activity to prevent aspecific pollen-tube growth and fertilization [109].

### 4.3. Transduction and Gene Regulation: Transcription Factors and Small Non Coding RNAs

The activation and expression pattern of SAGs are controlled at the levels of signal transduction, transcription and post-transcription [110].

G protein, secondary messenger calcium, polyamines and sugars signaling pathways were shown to induce floral senescence [76].

Before senescence starts, an increased level of G protein linked to the phospholipase a and c (PLA and PLC) was observed in *Petunia* petals: PLs hydrolyze the membrane component phosphatydylinositol diphosphate (PIP) to generate inositol triphosphate (IP3) and diacylglycerol (DAG), whose levels increase until the burst of ethylene [111].

As co-factor of protein kinases, the application of exogenous calcium in the orchid *Phalaenopsis* increased flower sensitivity to ethylene, thereby accelerating senescence [112].

In plants, polyamines (Pas) have roles in many physiological processes from the stimulation of cell division, differentiation and growth to stress response [113]. It has been shown that in *Dianthus caryophyllus* and gerbera petals the addition of different concentration of Pas, such as putrescine and spermine, caused a delay in senescence [114,115] probably inhibiting the ethylene synthesis. This is consistent with the results obtained by Lee *et al.* [116], where the application of a polyamine synthesis inhibitor on *Dianthus caryophyllus* petals led to increased levels of ACC synthase and aminocyclopropanecarboxylate oxidase (ACC oxidase) mRNAs as well as to increased ethylene production.

Finally, Hoeberichts *et al.*, in 2007 [117] reported that in *Dianthus caryophyllus* petals the application of soluble sugar and sucrose inhibited ethylene signaling with subsequent repression of SAGs and delay in protein degradation.

By means of genetic and transcriptomic approaches several plant transcription factors were identified as regulators of plant senescence. Most of the isolated transcription factors were involved in the positive regulation of leaf senescence. However, some *Arabidopsis* mutants such as NAC transcription factor (ANAC092) [118], AUXIN RESPONSIVE FACTOR 2 (ARF2) [119] and TCP4 [120] showed a delay in floral senescence suggesting their role in promoting floral abscission. Furthermore, the promoter of TCP4 and ANAC092 genes contains W-box motifs indicating a possible regulation by WRKY transcription factors. In particular, WRKY53 was identified as leaf senescence promoting transcription factor that also accelerates flowering [121].

In *Arabidopsis* the NAC-domain, bHLH and MYB73 genes were shown to be up-regulated soon after anthesis and their expression was restricted to ovule senescence [122].

In the senescing petals of *Dianthus caryophyllus* the Aux/IAA3 transcription factors were up-regulated [117], while MYB-like and a MYC proteins were differentially regulated although their function is unclear [117]. Four Ethylene insensitive 3 EIN3 and EIN3-like (EIL) transcription factors were isolated from *Dianthus caryophyllus* petals and showed differential expression levels, with the up-regulated EIL3 as key regulator of ethylene-responsive genes [117].

An AP2 protein with very high homology to ETHYLENE RESPONSIVE FACTOR 2 (ERF2) showed an up-regulation at transcription level in petals of *Narcissus* [96].

The expression levels of genes homologous to bZIP and HD-Zip proteins were up-regulated in *M. jalapa* flowers [84].

Three MADS box genes belonging to the AP1/AGL9 subfamily were isolated from orchid flowers [123]. Members of the same family in *Arabidopsis* were reported to be up-regulated and closely associated with flower and silique senescence [124]; the constitutive overexpression of the MADS domain protein AGAMOUS like 15 (AGL15) caused a delay in floral abscission in *Arabidopsis* [125].

The regulation of flower senescence was also shown to occur through epigenetic mechanisms as evident in the histone deacetylase *Arabidopsis* (*hda6*) mutant where the mutation induced a delay in floral senescence [126].

The regulation of gene expression occurs at the post-transcriptional level through the action of small non coding RNAs. Among them, the 21-nt microRNAs (miRNAs) exert important regulatory roles in plant development by silencing complementary mRNA transcripts. The miRNAs have been identified as regulators of flowering time and floral organ identity by controlling the expression of specific transcription factors. For example, in *Arabidopsis* the miR164 family silence members of the NAC genes with function in organ boundary formation [127] and the miR172 family regulates APETALA2(AP2) and other AP2-like transcription factor genes acting as repressors of flowering [128].

In *Arabidopsis* miR164 and miR319 families were shown to be involved in the control of leaf senescence [129]; although it has not yet been investigated, it is tempting to speculate that the same miRNA families might control flower senescence by regulating NAC and TCP transcription factors. Apart from ethylene and jasmonic acid, whose function was demonstrated to induce organ senescence, auxin plays a role in floral organ abscission through a miR390-TAS3-ARF2 module: miR390 induces the cleavage of a TAS3 transcript for the generation of 21-nt trans acting short interfering RNAs (tasiRNAs) involved in the silencing of ARF2, a negative regulator of auxin responses [119].

A recent study in *Rosa hybrid*: “*Vital*”, “*Maroussia*”, and “*Sympathy*” and *Rosa rugosa* Thunb. reported the identification of certain miRNAs putatively involved in the regulation of genes related to coloring, like the flavonoid biosynthetic genes [130]. In *Rosa* petals miR166 and miR5139 appeared to target β-galactosidase and expansin genes involved in cell-wall modification in an ethylene-regulated manner [131].

In *Arabidopsis* it was shown that by repressing the SQUAMOSA PROMOTER BINDING PROTEIN-LIKE 9 (SPL9) factor, miR156 regulates the FLOWERING LOCUS T (FT) expression during flowering and the accumulation of anthocyanin through the destabilization of a MYB-bHLH-WD40 transcriptional activation complex [132]: the high level of miR156 decreased the accumulation of anthocyanins and this result is consistent with the observed reduction of total anthocyanins concentration in petals [24,47] leading to a limited antioxidant defense and progress of senescence.

### 4.4. Antioxidant Genes

Antioxidant or ROS-scavenging genes have important roles in the control of ROS levels in different aspects of plant growth, development, response to stress and organ senescence. The increasing levels of reactive oxygen species (ROS) in senescing leaves and petals is accompanied by the differential regulation of genes involved in the protection against ROS. Transcriptome studies of leaves from *Arabidopsis* mutants, such as the *immutans* (*im*) variegation mutant [133], revealed that in response to oxidative stress the Cu-ZnSODs, FeSOD, CAT1, ferritin1, PODs, glutathione peroxidase (GPX2/GPX7), stromal APX, GR and alternative oxidase (AOX) genes were up-regulated whereas FeSOD1, CAT3, thylakoid APX and DHAR genes were down-regulated [134]. Interestingly, the work of Wagstaff *et al.* published in 2009 [135] showed that transcripts profiling of senescence in leaves, petals and siliques is similar, indicating that majority of SAGs are not tissue specific and supporting the hypothesis that petals are evolutionarily derived from leaves.

With respect to petal senescence large gene expression studies of CATs, APXs, lipoxygenases (LOX)s, SODs, GR and GSTs were performed in *Arabidopsis* [135], *Erysimum linifolium* [136], *Iris* [82] and *Alstroemeria* [137] and the expression patterns of some antioxidant genes were also identified through EST analysis in other species such as *Petunia* and *Dianthus caryophyllus*. Nevertheless, it is very likely that the expression pattern of antioxidant genes follows the same activity pattern of their encoded proteins, with a fall in the transcript abundances at the ethylene burst for ethylene sensitive species.

In *Alstroemeria* the expression levels of the CAT, APX, LOX and a Cu–Zn SOD genes were down-regulated and the DHAR gene was up-regulated, whereas in *Arabidopsis* these genes did not show variation in the mRNA abundance [50,137]. Members of the thioredoxin gene family were up-regulated in *Erysimum* and *Alstroemeria* [135,136,137]. Glutaredoxin genes and glutathione peroxidase were up-regulated in *Arabidopsis* and *Erysimum*, but down-regulated in *Alstroemeria*.

In *Dianthus caryophyllus*, two glutathione *S*-transferases (GST1, GST2) genes were up-regulated in concomitance with the increase of ethylene [138]. The functional role of most plant GSTs is still unclear, especially in petals: some act as glutathione peroxidises in *Arabidopsis*, while others might have roles in hormone metabolism [139] and anthocyanin biosynthesis [140].

An increased transcript level of metallothioneins (MTs) was reported in senescent leaves [141] petals [83], and ripening fruits [142]. MTs were highly represented in the transcriptome of *Alstroemeria* and in *Erysimum* senescent petals but not in *Arabidopsis* [50,81,137]. The function of such high levels of MTs might be related to the detoxification of metal ions (Cu^2+^ and Zn^2+^) released after enzyme and protein degradation.

During the initial stages of senescence the antioxidant response to oxidative stress is associated with an increment in the content of antioxidant compounds such as tocopherol, ascorbic acid (AsA) and glutathione, followed by a decrease as senescence reaches the ultimate phases.

The key genes in tocopherol biosynthesis were isolated such as the HPD encoding for 4-hydroxyphenylpyruvate dioxygenase and VTE2 encoding for homogentisate prenyltransferase. HPD expression analysis in rice and barley leaves showed that the mRNA levels were enhanced during leaf senescence [143] and tocopherols were found to be accumulated in the senescing leaves of several plants [144]. In particular, an increasing level of α- and γ-tocopherol were observed during senescence of *Lilium* cut flowers [145].

Ascorbic acid is synthetized via l-galactose [146], l-gulose [147], d-galacturonic and d-glucuronic pathways [148]. The genes encoding the biosynthetic enzymes were identified through the study of four AsA-deficient mutants (*vtc1*, *vtc2*, *vtc3*, and *vtc4*) [149]. As a result of the decreased enzyme activity, the *vtc1* mutant had a reduced quantity of AsA respect to the wild-type [150]. Kotchoni *et al.* in 2009 [151] reported that low levels of AsA induced the up-regulation of circadian clock and photoperiodic pathway genes and the down-regulation of FLC, causing early flowering, while high levels of AsA delayed flowering and senescence irrespective of the photoperiod.

Mutants showing alterations in flower pigmentation were affected in the flavonoid biosynthesis and many of the involved regulatory genes were isolated from *Arabidopsis*, *Zea mais*, *Antirrhinum majus*, and *Petunia* [152]. The accumulation of anthocyanin depends on the expression level of chalcone synthase (CHS), chalcone isomerase (CHI), phenylalanine ammonia-lyase (PAL) and flavonoids 3′-hydroxylase (F3′H) biosynthetic genes. Their expression increases during the early stages of flower development and decreases at the later stages in *Petunia*, *Malus domestica*, *Antirrhinum majus* and *Rose*.

Interestingly, the overexpression of a GAST-like protein (GIP2) in *Petunia* promoted stem and corolla elongation by regulating the levels of ROS, acting as antioxidant [153].

## 5. Conclusions

With the advancement in sequencing technologies and transcriptome profiling tools, the global gene expression reprogramming of floral senescence has been elucidated in diverse species despite the function of many genes has yet to be validated. Among plant species, there are evident differences in the transcript levels of SAGs and antioxidant genes: these may be ascribed to the diversity of mechanisms established during senescence. Such differences could also be due to the variation of SAGs and antioxidant transcript levels among different cells that might undergo stages of senescence at different times within the same floral tissue.

Hence, still many aspects of floral senescence have to be deciphered. Firstly, a more comprehensive profiling of the small RNAs classes is necessary to better understand the gene regulatory mechanisms at the post-transcriptional level. Secondly, little information is available for abiotic stresses impacts on flower development and senescence. For example, a large variability exists in the senescence response to climate change depending on the climatic conditions such as temperature and on the plant species [154]. Both proteomic and metabolomics investigations are needed to determine the post-translational modifications and the metabolic changes. The combination of different “omics” platforms will surely provide a global and deeper view of all the networks implicated in flower development and senescence.

Despite the difficulty in transforming and regenerate certain species, the understanding of senescence regulatory networks will help to engineer flowers with an extended life. It was shown that *Dianthus caryophyllus* flowers expressing an antisense copy of ACC oxidase (ACO) or the expression of ethylene insensitive receptor etr1-1 in *Petunia* showing delay in floral abscission had an increased longevity respect to the wild type and to the application of any postharvest treatment [155,156]. Genetic engineering might be also addressed toward the manipulation of antioxidant gene expression and enzymes involved in antioxidants production pathway (*i.e.*, ascorbic acid, polyphenols, *etc.*) both in ornamental flowers and edible flowers, the latter with direct positive implications for human nutrition and health. Senescence conditions generally determine an increase in antioxidant levels as they represent a key defense strategy against oxidative stress. As such these phases could be very interesting on both nutritional and ornamental point of view. Moreover, studies on transcription factors and genes responsible for antioxidant-related traits will be a valid support in the identification of varieties with a better attitude to storage and industrial processing as well as in the molecular assisted selection.

Further works should be addressed on the quality of edible flowers after harvest and the development of technologies that can be used for maintaining the nutritional value during storage. The marketability of edible flowers will be possible if appropriate food processing and storage methods will be studied and designed in order to reduce quality losses from the field to the consumers. Only if an organized food chain will be developed the ornamental flower will gain the global market. Postharvest treatments should be also optimized for these new foods considering that only safe and non-toxic compounds can be utilized for the storage of edible products.
